# Age-Related Impairment of Innate and Adaptive Immune Responses Exacerbates Herpes Simplex Viral Infection

**DOI:** 10.3390/pathogens14070624

**Published:** 2025-06-23

**Authors:** Ruchi Srivastava, Sweta Karan, Yassir Lekbach, Afshana Quadiri, Ava Tohidian, Chhaya Maurya, Sarah Xue Le Ng, Reilly Chow, America Garcia, Anshu Agrawal, Hawa Vahed, Aziz A. Chentoufi, Lbachir BenMohamed

**Affiliations:** 1 Laboratory of Cellular and Molecular Immunology, Gavin Herbert Eye Institute, School of Medicine, University of California Irvine, Irvine, CA 92697, USA; ruchisgpgi@gmail.com (R.S.); ylekbach@hs.uci.edu (Y.L.); avagtohidian@gmail.com (A.T.); cmaurya@uci.edu (C.M.); reillyac@uci.edu (R.C.); amerig2@uci.edu (A.G.); hvahed@hs.uci.edu (H.V.);; 2 Division of Basic and Clinical Immunology, Department of Medicine, University of California Irvine, Irvine, CA 92697-4375, USA; aagrawal@hs.uci.edu; 3 Department of Pathology, University of California Irvine, Irvine, CA 92697-4375, USA; 4 Department of Molecular Biology & Biochemistry, Institute for Immunology, School of Medicine, University of California Irvine, Irvine, CA 92697, USA; 5 Department of Vaccines and Immunotherapies, TechImmune, LLC, University Lab Partners, Irvine, CA 92660-7913, USA

**Keywords:** herpes, aging, DCs, CD8^+^ T cells

## Abstract

Immune function declines with age, leading to increased vulnerability of the elderly to viral infectious pathogens. The mechanisms by which aging negatively impacts the innate and adaptive immune system, leading to enhanced susceptibility to respiratory viral pathogens, remain incompletely understood. In the present study, we utilized a mouse model of infection with herpes simplex virus type 1 (HSV-1), a virus that can infect the lungs and lead to pneumonia, a rare but serious health concern in the elderly. Following intranasal inoculation of young (6 weeks), adult (36 weeks), and aged mice (68 weeks) with HSV-1 (KOS strain) we: (i) compared the local and systemic immune responses to infection in young, adult, and aged mice, and (ii) correlated the level and type of immune responses to protection against HSV-1 infection and disease. Compared to young and adult mice, aged mice displayed: (i) increased activation of epithelial cells with a decreased expression of TLR3; (ii) increased activation of dendritic cells with increased expression of MHC-I, MHC-II, and CD80/86; (iii) decreased production of type-I interferons; (iv) delayed production of anti-inflammatory cytokines and chemokines in the lungs; and (v) impairment frequencies of functional HSV-specific CD107+IFN-γ+CD8+ T cells associated with the increased incidence of viral infection and disease. These findings suggest that age-related impairments in innate and adaptive immune responses may exacerbate respiratory viral infections and disease in the elderly.

## 1. Introduction

Herpes simplex virus type 1 (HSV-1) causes various infections that involve mucocutaneous surfaces, such as the genital tract, the cornea, the central nervous system, and, occasionally, visceral organs, including the lungs [1,2]. Infection of the respiratory tract with HSV can cause herpes simplex pneumonia (HSP). Although HSV-1 pneumonia is rare, especially since antivirals are sufficient to treat the disease, the inciting virus enters the lung via the hematogenic route during viremia. Immune function declines with age, making elderly individuals more susceptible to pulmonary viral infections. Seventy percent of the human population infected with HSV are adults above the age of 65.

The virus has been reported to be associated with pulmonary disease since 1949 [3]. Pulmonary innate immune responses rely on a highly regulated multicellular network to defend an enormous surface area of interaction with the external world. Local disruption of these responses renders the host susceptible to respiratory infections and facilitates the systemic spread of infection. Studies over the last decade have determined that airway epithelial cells, dendritic cells (DCs), and macrophages are key participants in this innate immune network. DCs are the major antigen-presenting cells that can sense and respond to pathogens and activate T cells in the lungs.

Peripheral immune responses are virtually constant in the young but are invariably reduced in aged mice and humans. The increased morbidity and mortality reported in elderly populations are due to several factors, including dysfunctions in the senescent immune system. Several in vitro and in vivo studies have pointed to the type I interferon (IFN) response as a critical pathway involved in the early immune response to infection [4,5,6]. Multiple pattern recognition receptors, such as the toll-like receptor 3 (TLR-3), have also been demonstrated to be involved in controlling viral replication in the murine models of infection [7].

As the elderly population continues to increase worldwide, a deeper understanding of the changes that the immune system undergoes during the aging process is becoming a key factor in the development of new therapeutic strategies. Given the decline in the immune function of the elderly, this puts them at a high-risk population for clinical herpes virus reactivation and/or severe disease; therefore, new therapeutic targets are urgently needed. HSV-1 has been reported to infect the respiratory tract, mainly in immunocompromised and elderly individuals, leading to complications such as bronchitis and even acute respiratory distress syndrome [8,9]. These age-associated immune deficits contribute to increased vulnerability to HSV-1-associated respiratory viral infections [10]. Therefore, the choice of HSV-1 as a model for respiratory infection is highly relevant for studying age-related respiratory pathogenesis. Additionally, associations between innate and adaptive immune responses of the elderly population have not been fully examined in herpes simplex infection and disease. Therefore, we explored the age-related immune cell responses (in terms of magnitude, functional capacity, and repertoire diversity) in young mice (6 weeks of age), adult mice (36 weeks of age), and aged mice (68 weeks of age) following intranasal herpes simplex virus type 1 (HSV-1) infection. After infection with the herpes simplex virus, a significantly reduced T cell response was observed in old compared to young mice. More importantly, control of virus replication was profoundly impaired in aged mice compared to young mice. We evaluated age-specific changes in dendritic cell function, cytokine production, and T cell activation within the respiratory mucosa, a critical site for host–pathogen interaction. In summary, our results demonstrate an impaired immune response to herpes infection in aged mice compared to young mice, further supporting the concept of immunosenescence in the elderly.

## 2. Materials and Methods

***Mice:*** Six-week-old (young), 36-week-old (adult), and 68-week-old (aged) female C57BL/6 (B6) mice were purchased from The Jackson Laboratory (Bar Harbor, ME, USA). Based on well-established murine-to-human age conversion models, the average median lifespan of a laboratory mouse is approximately 28 months [11]. The 6-week-old mice correspond to ~4-year-old young adults in humans, 36-week-old mice to middle-aged adults, ~27 years old, and 68-week-old mice to older adults, ~50–55 years old [11]. These time-points were specifically chosen to model age-related immune decline and its impact on HSV-1 infection and associated pathology. Animal studies conducted at the University of California conformed to the Guide for the Care and Use of Laboratory Animals published by the US National Institute of Health (IACUC protocol # AUP 19-111).

***Virus production:*** Herpes simplex virus type 1 (HSV-1, strain KOS) was grown and titrated on rabbit skin (RS) cells as previously described [12,13,14,15].

***Intranasal infection****:* All three groups of mice were anesthetized with intraperitoneal injection of a mixture of ketamine and xylazine, at the dose 87.5 mg/kg Ketamine and 12.5 mg/kg Xylazine, and then intranasally infected with 50 μL of PBS containing 1 **×** 10^6^ pfu of HSV-1 (KOS strain).

***Nasal swab collection:*** Nasal swabs were collected on day 2 and day 6 post-infection by pipetting 30 µL of phosphate-buffered saline (PBS) in and out of the nasal openings three times and were frozen at−80 °C.

***Viral titer assay:*** Nasal washes and lung tissue were analyzed for viral titers by plaque assays. Vero cells were grown in an α-modified Eagle’s medium (ThermoFisher Scientific, Waltham, MA, USA) supplemented with 5% fetal bovine serum, 1% penicillin-streptomycin, and L-glutamine (ThermoFisher Scientific, Waltham, MA, USA). For plaque assays, Vero cells were grown to confluence in 24-well plates. Nasal wash samples were added to monolayers. Infected monolayers were incubated for 1 h at 37 °C and rocked every 15 min to facilitate viral absorption. Infected monolayers were overlaid with media containing carboxymethyl cellulose. Infection was allowed to occur for 72 h at 37 °C. Monolayers were then fixed and stained with crystal violet. Then the viral plaques were counted under a light microscope. Positive controls were run with every assay with previously titrated laboratory stocks of HSV-1.

***Tissue Harvesting and Lung Cell Isolation:*** Mice were anesthetized with an intraperitoneal injection of ketamine/xylazine at a of 87.5 mg/kg Ketamine and 12.5 mg/kg Xylazine. For euthanasia, the anesthetized mice were exposed to carbon dioxide inhalation followed by cervical dislocation. Lungs were harvested (*n* = 5 per time point per experiment) on days 2 and 6 post-infection. Lungs were exposed by opening the chest cavity and rinsed with cold 1× PBS through the right heart ventricle. Mice lung tissues were harvested, minced, and digested in 5 mg/mL collagenase for 45 minutes before filtering through a 70 µm cell strainer. Subsequently, the cells were centrifuged and diluted to 1 × 10^6^ viable cells/mL in RPMI medium supplemented with 10% (*v*/*v*) fetal bovine serum (FBS). Viability was determined by Trypan blue staining.

***Flow cytometry****:* Single-cell suspensions from the lungs were prepared for flow cytometric analysis. The following antibodies were used: anti-mouse CD8 PerCP (BD Biosciences, San Jose, CA, USA), anti-mouse CD11b FITC (BD Biosciences), anti-mouse CD103 APC (BD Biosciences, San Jose, CA, USA), anti-mouse CD11c (BD Biosciences, San Jose, CA, USA), anti-mouse CD45 APC-cy7 (BioLegend, San Diego, CA, USA), anti-mouse TLR3 (BD Biosciences), anti-mouse MHCI (BD Biosciences), MHCII (eBioscience, San Diego, CA, USA), CD4 PerCP, CD107^a^ FITC, CD107^b^ FITC (BD Biosciences), and anti-mouse IFN-γ PE-cy7 (clone XMG1.2, BioLegend, San Diego, CA, USA). For surface staining, monoclonal antibodies (mAbs) were added against various cell markers to a total of 1 × 10^6^ cells in phosphate-buffered saline (PBS) containing 1% fetal bovine serum (FBS) and 0.1% Sodium azide (fluorescence-activated cell sorter [FACS] buffer), and left for 45 min at 4 °C. For intracellular staining, cells were first treated with Cytofix/Cytopperm (BD Biosciences) for 30 min BioLegend, San Diego, CA, USA. Upon washing with Perm/Wash buffer, mAbs were added to the cells and incubated for 45 min on ice in the dark. Cells were subsequently washed with Perm/Wash and FACS buffer and fixed in PBS containing 2% paraformaldehyde (Sigma-Aldrich, St. Louis, MO, USA).

For the measurement of CD107^a/b^ and IFN-γ, 1 × 10^6^ cells were first transferred into a 96-well flat-bottom plate in the presence of BD GolgiStop (10 μg/mL) for 6 h at 37 °C. Phytohemagglutinin (PHA) (5 μg/mL) (Sigma-Aldrich) was used as a positive control. At the end of the incubation period, the cells were transferred to a 96-well round-bottom plate and washed once with FACS buffer. Surface and intracellular staining were performed as previously described. A total of 100,000 events were acquired by the LSRII (Becton Dickinson, Mountain View, CA, USA), followed by analysis using the FlowJo software.10.4.1 (TreeStar, Ashland, OR, USA).

***Cytokine Analysis:*** Lung cells collected on days 2 and 6 post-infection were cultured overnight, and the supernatants were assayed for cytokines and chemokines using the multiplex magnetic bead-based kit (ThermoFisher Scientific, Waltham, MA, USA).

***Statistical analysis:*** We examined the distribution of each immunological parameter [12]. In the case of two-group comparisons, we considered the use of the parametric two-sample Student’s *t*-test or non-parametric Wilcoxon rank sum test. In addition, for paired comparisons involving multiple peptides, we have adjusted for multiple comparisons using the Bonferroni procedure. In the specific case of three groups (comparing two subgroups with a baseline subgroup), we used the General Linear Model procedure and compared the least squares means using the Dunnett procedure for multiple comparisons. Flow cytometry data were analyzed with analyzed using FlowJo software v10.10 (Becton Dickinson, Ashland). SAS^®^ v.9.4 (Statistical Analysis System, Cary, NC, USA) was used for analysis. Graphs were prepared with GraphPad Prism version 10.4.1 software (San Diego, CA, USA). Data are expressed as the mean ± SD. Results were statistically significant at *p* < 0.05.

## 3. Results

### 3.1. Decreased TLR-3 Expression and Increased Activation of Airway Epithelial Cells (AECs) in Aged Mice at Homeostasis

**Figure 1:**
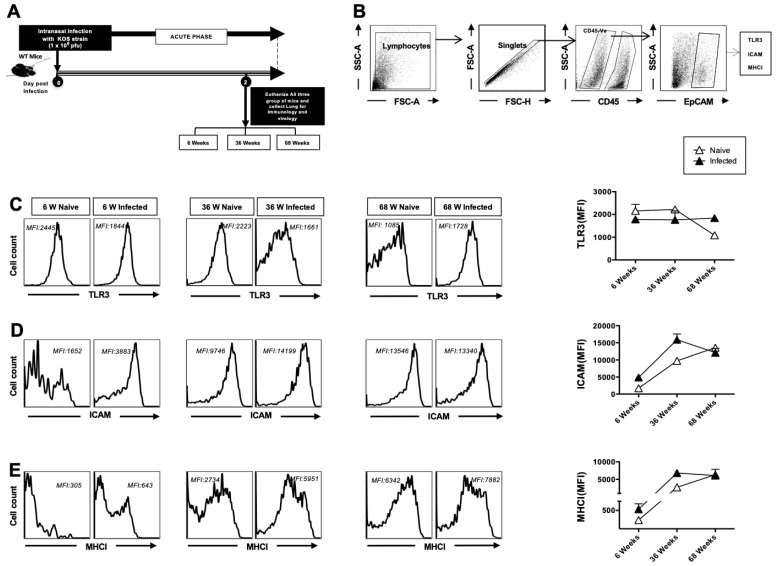
TLR3 expression and activation of airway epithelial cells (AECs): 6-week-old, 36-week-old, and 68-week-old mice were infected intranasally with 1 × 10^6^ pfu of HSV-1 strain. The mice were euthanized 2 days post-infection and a single cell suspension from the lungs was obtained after collagenase treatment. The lung cells were stained for epithelial cell markers, TLR-3, and activation markers, and then analyzed by flow cytometry (FACS). (**A**) Timeline of infection and immunological analyses. (**B**) Gating strategy used to characterize lung-derived cells. Lymphocytes were identified by a forward scatter (FSC) and side scatter (SSC) gate. Singlets were selected by plotting forward scatter area (FSC-A) vs. forward scatter height (FSC-H). CD45-negative cells were then gated. The epithelial cell population in CD45-gated cells was defined by the EpCAM^+^ cells. (**C**) Representative histograms and average mean fluorescence intensity (MFI) of TLR-3 expression on the surface of lung epithelial cells. (**D**) ICAM expression on epithelial cells. (**E**) MHCI expression on lung epithelial cells of 6-week-old, 36-week-old, and 68-week-old mice at 2 days post-infection with HSV-1 (solid black) or mock-infection with DMEM (solid white). The data represent two separate experiments. Graphs depict the mean +/− S.E. of 5 mice/group. Results were statistically significant at *p* ≤ 0.05.

Airway epithelial cells (AECs) are the first cells to sense and respond to infections [16]. Viral replication also primarily occurs in the epithelium. Herpes infection signals via TLR3 to elicit antiviral innate immune responses in host cells [17]. TLR3 is a pattern recognition receptor that recognizes viral double-stranded RNA produced during viral replication, including that of HSV-1 [18]. Herein, we studied the role of AEC responses in the innate immune defense against intranasal infection with HSV-1. Mice aged 6 (*n* = 5), 36 (*n* = 5), and 68 (*n* = 5) weeks were infected intranasally with 1 × 10^6^ pfu of HSV-1 strain (KOS). Two days post-infection, the mice were euthanized, and a single-cell suspension from the lungs was obtained after collagenase treatment. CD45-EPCAM^+^ AECs were analyzed for the expression of TLR3, ICAM-1 (CD54), and MHC-I via flow cytometry (Figure 1A,B). AECs from aged mice displayed reduced expression of TLR3 at homeostasis as compared to adult and young mice, indicating a reduced capacity to sense HSV-1 infection (Figure 1C). In contrast, the expression of ICAM-1 and MHC-I in AECs was higher in aged mice at homeostasis. Since these are markers of activated epithelium, our results suggest that the airway epithelium appeared to be inherently activated at baseline, in the absence of infection (Figure 1D,E). Upon infection, the expression of ICAM-1 and MHC-I was upregulated in infected young and infected adult mice but remained unchanged in infected aged mice, suggesting that aged AECs did not respond to HSV-1 infection (Figure 1D,E). Taken together, these results indicate that AECs from aged mice are impaired in their capacity to sense and respond to infections, and display an increased basal level of epithelial activation.

### 3.2. The Expression of TLR3 on Dendritic Cells and Macrophages in the Lungs Decreases with Age at Homeostasis

**Figure 2:**
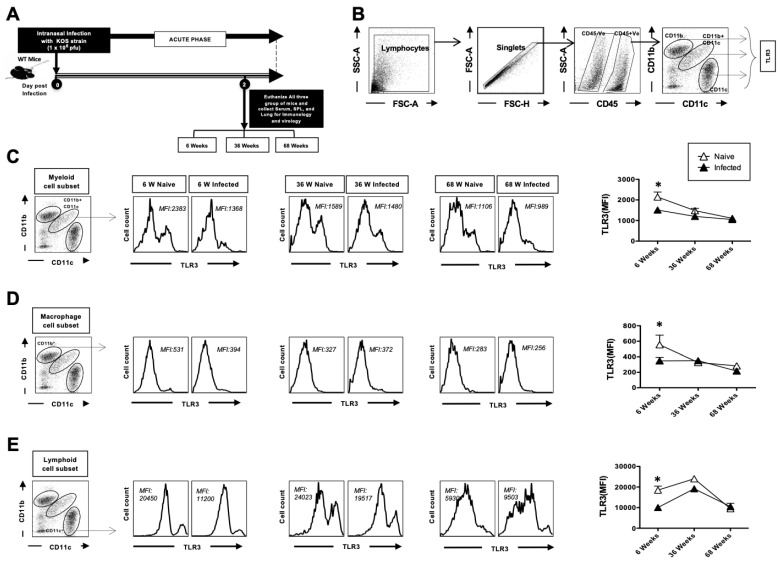
The expression analysis of TLR3 on DCs and macrophages in the lungs: 6-week-old, 36-week-old, and 68-week-old mice were infected intranasally with 1 × 10^6^ pfu of HSV-1 strain. Two days post-infection, the mice were euthanized, and a single-cell suspension from the lungs was obtained after collagenase treatment. The lung cells were stained for dendritic cell markers and activation markers, and then analyzed by flow cytometry (FACS). (**A**) Timeline of infection and immunological analyses. (**B**) Gating strategy used to characterize lung-derived cells. Lymphocytes were identified by a forward scatter (FSC) and side scatter (SSC) gate. Singlets were selected by plotting forward scatter area (FSC-A) vs. forward scatter height (FSC-H). CD45-positive cells were then gated based on their expression of CD45. The myeloid cell population in CD45^+^ gated cells was defined by the CD11b^+^ CD11c^+^ cells. Similarly, the CD11b^+^ subset was defined as macrophages, and the CD11c^+^ cells as lymphoid cell subsets. Representative histograms and average mean fluorescence intensity (MFI) of TLR-3 expression on the surface of (**C**) myeloid cell subset (CD11b^+^CD11c^+^), (**D**) macrophages (CD11b^+^CD11c^−^), and (**E**) lymphoid DC (CD11c^+^) from the 6-week-old, 36-week-old, and 68-week-old mice at 2 days post-infection with HSV-1 (solid black) or mock-infection with DMEM (solid white). The data represent two separate experiments. Graphs depict the mean +/− S.E. of 5 mice/group. * Results were statistically significant at *p* ≤ 0.05.

Next, we examined the expression of TLR3 on DCs and macrophages, since these cells also get activated via TLRs (Figure 2A). The single-cell lung suspension prepared from the lungs (Figure 1) was also utilized for these experiments. To elucidate the consequences of aging on TLR expression in various populations of DCs, we utilized multicolor flow cytometry staining in lung samples from young, adult, and aged mice (Figure 2B); lymphocytes were identified by a forward scatter (FSC) and side scatter (SSC) gate. Singlets were selected by plotting forward scatter area (FSC-A) vs. forward scatter height (FSC-H). CD45-positive cells were then gated based on their expression of CD45. The myeloid cell population in CD45^+^ gated cells was defined by the CD11b^+^ CD11c^+^ cells (mDC). Similarly, the CD11b^+^ subset was defined as macrophages, and the CD11c^+^ cells as lymphoid cell subsets. TLR-3 expression was analyzed on the surface of myeloid cell subsets (CD11b^+^CD11c^+^), macrophages (CD11b^+^CD11c^−^), and lymphoid dendritic cells (CD11c^+^) from 6-, 36-, and 68-week-old mice at 2 days post-infection. As shown in Figure 2C–E, the results demonstrated that TLR-3 expression was decreased at baseline in 68-week-old mice as compared to adult and young mice. The difference between the groups was not significant after infection. Decreased TLR3 expression at baseline in DCs and macrophages from aged mice is indicative of their compromised ability to sense and respond to TLR3 ligands, including HSV-1, making them more susceptible to these viral infections.

### 3.3. The Upregulation of MHC-I After Herpes Infection Is Impaired with Age

**Figure 3:**
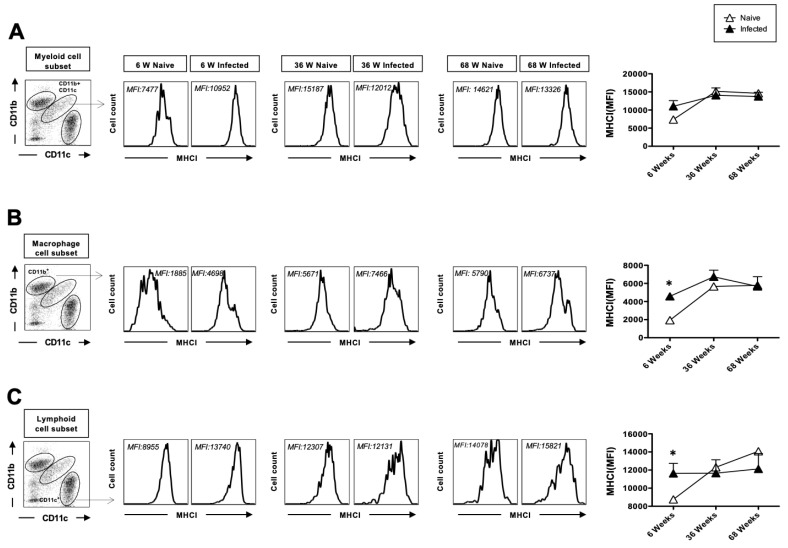
The upregulation of MHC-I after herpes infection: 6-week-old, 36-week-old, and 68-week-old mice were infected intranasally with 1 × 10^6^ pfu of HSV-1 strain. Two days post-infection, the mice were euthanized, and a single cell suspension from the lungs was obtained after collagenase treatment. The lung cells were stained for dendritic cell markers and activation markers, and then analyzed by flow cytometry (FACS). CD45-positive cells were then gated based on their expression of CD45. The myeloid cell population in CD45^+^ gated cells was defined by the CD11b^+^ CD11c^+^ cells. Similarly, the CD11b^+^ subset was defined as macrophages, and the CD11c^+^ cells as lymphoid cell subsets. Representative histograms of MHC-I expression mean fluorescence intensity (MFI) on the surface of (**A**) myeloid cell subset (CD11b^+^CD11c^+^), (**B**) macrophages (CD11b^+^CD11c^−^), and (**C**) lymphoid DC (CD11c^+^) from 6-week-old, 36-week-old, and 68-week-old mice at 2 days post-infection with HSV-1 (solid black) or mock-infection with DMEM (solid white). The data represent two separate experiments. Graphs depict the mean +/− S.E. of 5 mice/group. * Results were statistically significant at *p* ≤ 0.05.

Since DCs and macrophages are antigen-presenting cells, we examined the upregulation of MHC class I and MHC class II cell surface markers on these cells. The upregulation of MHC-I on DC, as the primary antigen-presenting cells, is required for priming CD8^+^ T cell responses. The surface expression levels of MHC-I were assessed on lung mDCs, macrophages, and lymphoid DCs collected from young (*n* = 5), adult (*n* = 5), and aged (*n* = 5) mice. Only the aged mice exhibited upregulated levels of MHC-I on lung mDCs, macrophages, and lymphoid DCs at baseline without infection. Two days post-infection, a significant upregulation of MHC-I was observed in lung mDCs, macrophages, and lymphoid DCs from young mice (Figure 3A–C). In contrast, MHC-I levels in adult and aged mice remained essentially unchanged post-infection. However, both naïve and infected conditions in these groups showed higher overall MHC-I expression than in young mice. Notably, MHC-I expression appeared to plateau with age in both mDCs and macrophages (Figure 3A,B), while lymphoid DCs showed age-dependent increases at baseline (Figure 3C).

### 3.4. The Upregulation of MHC-II After Herpes Infection Is Impaired with Age and Exhibits Reduced Secretion of Anti-Inflammatory Cytokines in the Lungs After Acute HSV Infection

**Figure 4:**
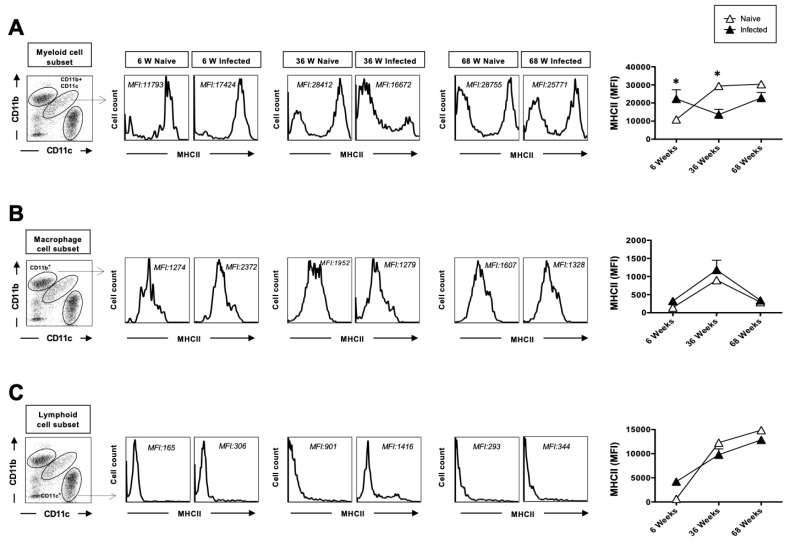
The upregulation of MHC-II after herpes infection: 6-week-old, 36-week-old, and 68-week-old mice were infected intranasally with 1 × 10^6^ pfu of HSV-1 strain. Two days post-infection, the mice were euthanized, and a single-cell suspension from the lungs was obtained after collagenase treatment. The lung cells were stained for dendritic cell markers and activation markers, and then analyzed by flow cytometry (FACS). CD45-positive cells were then gated based on their expression of CD45. The myeloid cell population in CD45^+^ gated cells was defined by the CD11b^+^ CD11c^+^ cells. Similarly, the CD11b^+^ subset was defined as macrophages, and the CD11c^+^ cells as lymphoid cell subsets. Representative histograms mean fluorescence intensity (MFI) of MHC-II expression on the surface of (**A**) myeloid cell subset (CD11b^+^CD11c^+^), (**B**) macrophages (CD11b^+^CD11c^−^), and (**C**) lymphoid DC (CD11c^+^) from 6-week-old, 36-week-old, and 68-week-old mice at 2 days post-infection with HSV-1 (solid black) or mock-infection with DMEM (solid white). The data represent two separate experiments. Graphs depict the mean +/− S.E. of 5 mice/group. Results were statistically significant at *p* ≤ 0.05. Graphs depict the mean +/− S.E. of 5 mice/group. * Results were statistically significant at *p* ≤ 0.05.

We next examined the effect of MHC-II expression on dendritic cells (DCs) and macrophages. DCs and macrophages, upon activation, upregulate MHC-II expression, enabling them to present antigens to CD4^+^ T cells and therefore activate them. Both adult and aged mice exhibited upregulated levels of MHC-II on lung myeloid dendritic cells (mDCs), macrophages, and lymphoid dendritic cells at baseline, prior to HSV-1 infection. Cell surface expression of MHC II on lung mDCs was significantly upregulated within 2 days post-infection in young mice but did not increase on the same cells in adult and aged mice (Figure 4A). MHC class II expression was similar on lung macrophages, although adult mice displayed higher MHC-II expression at baseline and post-infection compared to young and aged mice (Figure 4B). Lymphoid DCs displayed a similar pattern of MHC-II expression as mDCs (Figure 4C).

### 3.5. Aged Mice Display Reduced Secretion of Anti-Inflammatory Cytokines in the Lungs After Acute HSV Infection

**Figure 5:**
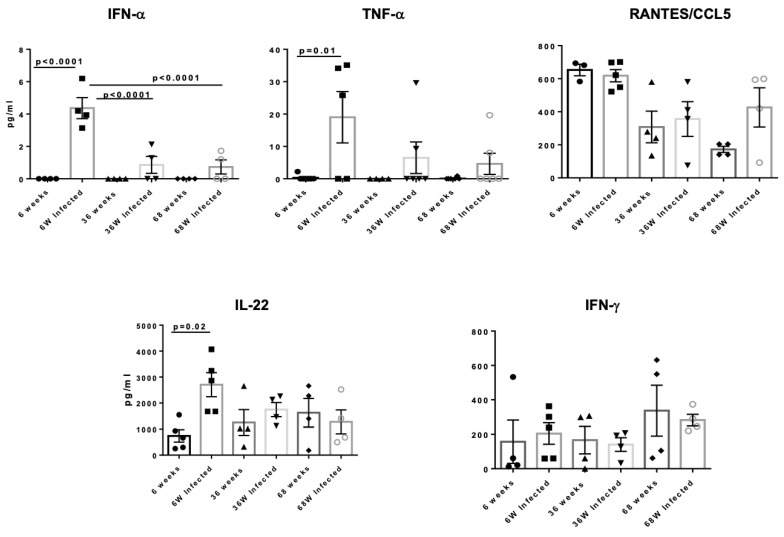
Analysis of anti-inflammatory cytokines in the lungs after acute HSV infection. Aged mice display reduced secretion of protective cytokines in the lungs after acute HSV infection. Multiplex detection was used to quantify the levels of inflammatory mediators in the lungs of 6-week-old, 36-week-old, and 68-week-old mice on day 2 post-infection. The data represent two separate experiments. Graphs depict the mean +/− S.E. of 5 mice/group. Results were statistically significant at *p* ≤ 0.05.

Cytokines play a crucial role in establishing an antiviral state as the nonspecific first line of defense against viral infections. Changes due to aging occur across a wide range of immune parameters, including the induction of cell surface activation markers, cytokine secretion, and proliferative capacity. In this study, we investigated the impact of age on the production of interferon (IFN)- α, TNF-α, RANTES, IL-22, and IFN-γ. We utilized a multiplex detection method to quantify the levels of inflammatory mediators in the lungs of mice aged 6 weeks (*n* = 5), 36 weeks (*n* = 5), and 68 weeks (*n* = 5) on day 2 post-infection. The aforementioned cytokines and chemokines were measured in total lung tissue. We detected that IFN-α and TNF- α expression was increased significantly in young mice after herpes infection, whereas there was no change in adult and aged mice indicating reduced response to infection with age (Figure 5). Both IFN-α and TNF- α play an important role in the induction of anti-viral CD4 and CD8 responses. The cytokine, IL-22, also displayed a significant increase in young mice post-infection with no change in adult and aged mice. This is important, as IL-22 has been reported to protect mice from virus-induced lung injury [19,20]. No difference was found between RANTES/CCL5 and IFN-γ. Increased levels of CCL5 were observed in an aged, infected group compared to the uninfected group. These results suggest that aged mice exhibit a deficiency in the production of antiviral and protective cytokines, which may contribute to their increased susceptibility to infections.

### 3.6. Viral Replication in the Lungs and Disease Severity Increase with Age

**Figure 6:**
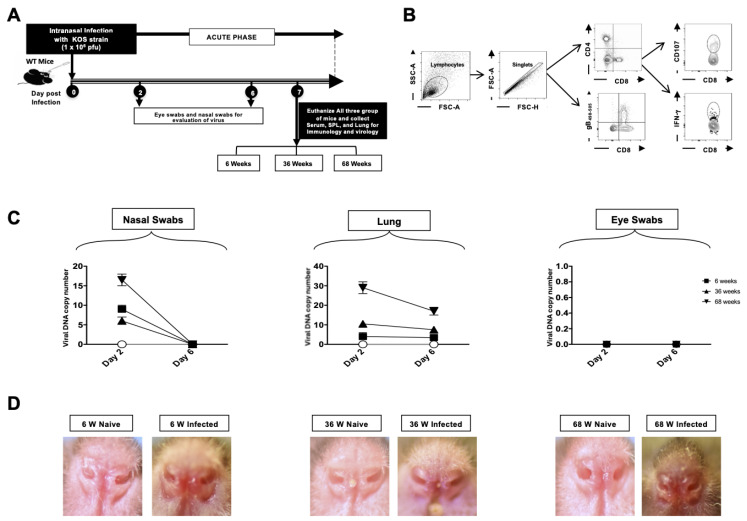
Viral titers and severity of HSV lung infection: 6-week-old, 36-week-old, and 68-week-old mice were infected intranasally with 1 × 10^6^ pfu of HSV-1 strain. The mice were euthanized on day 6 post-infection, and a single cell suspension from the lungs was obtained after collagenase treatment. The lung cells were stained for T-cell markers and then analyzed by flow cytometry (FACS). (**A**) Timeline of infection and immunological analyses. (**B**) Gating strategy used to characterize lung-derived cells. Lymphocytes were identified by a forward scatter (FSC) and side scatter (SSC) gate. Singlets were selected by plotting forward scatter area (FSC-A) vs. forward scatter height (FSC-H). CD8 and CD4 cell-positive cells were then gated based on the expression of CD8 and CD4 antibodies. (**C**) Nasal swabs and eye swabs were collected from 6-week-old, 36-week-old, and 68-week-old mice on days 2 and 6 for viral DNA copy numbers to be measured in the nasal swabs, lungs, and eye swabs on days 2 and 6 post-infection. (**D**) Representative nostril images of naïve and infected 6-week, 36-week, and 68-week-old mice. The data represent two separate experiments. Graphs depict the mean +/− S.E. of 5 mice/group. Results were statistically significant at *p* ≤ 0.05.

The mice were inoculated intranasally with HSV-1 (KOS strain). Nasal swabs and eye swabs, and lung tissues were collected from 6-week-old (*n* = 5), 36-week-old (*n* = 5), and 68-week-old (*n* = 5) mice on day 2 and day 6 for viral titer estimation. An illustration of the infection scheme and the timeline of subsequent immunological and virological assays is shown in Figure 6A. The gating strategy for T cells is shown in Figure 6B; lymphocytes were identified by a forward scatter (FSC) and side scatter (SSC) gate. Singlets were selected by plotting forward scatter area (FSC-A) vs. forward scatter height (FSC-H). CD4+ and CD8+ cells were then gated based on the expression of CD4 and CD8. The DNA copy numbers were highest in the nasal swab and lung tissue of 68-week-old mice as compared to 36-week-old and 6-week-old mice at day 2 (Figure 6C). By day 6, infection was controlled, and viral DNA loads were down across all age groups. In keeping with the titers, 68-week-old infected mice displayed the highest lesions, followed by 6-week-old mice, while 36-week-old mice had moderate lesions (Figure 6D). The virus was undetectable in the eye swabs. Altogether, this data indicates that HSV lung infection severity increases in aged mice.

### 3.7. The Generation of Functional HSV-Specific CD8^+^ T Cells Is Impaired in Aged Mice Following Intranasal Infection with HSV-1

**Figure 7:**
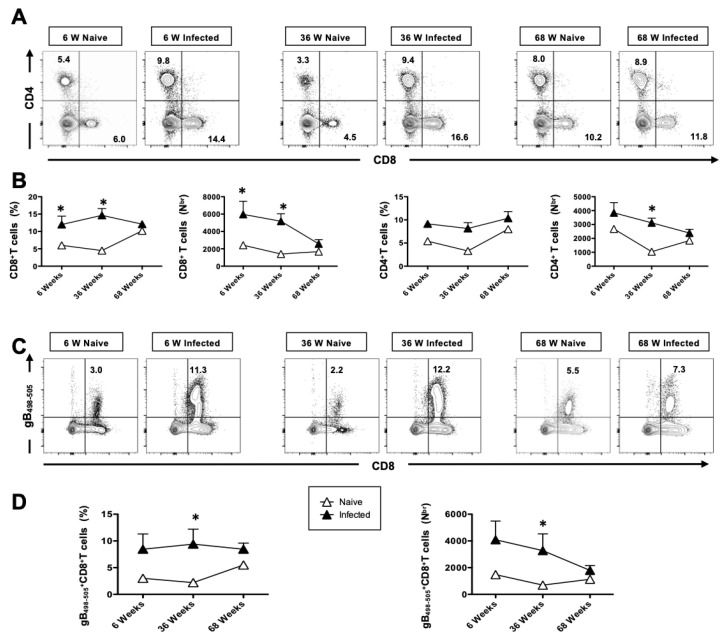
The generation of HSV-specific tetramer-positive CD8^+^ T cells: 6-week-old, 36-week-old, and 68-week-old mice were infected intranasally with 1 × 10^6^ pfu of HSV-1 strain. Seven days post-infection, the mice were euthanized, and a single-cell suspension from the lungs was obtained after collagenase treatment. The lung cells were stained for T cell markers and then analyzed by FACS. (**A**) Representative FACS plots of the frequencies of CD4^+^ and CD8^+^ T cells detected in the lungs of HSV-1-infected and mock-infected control groups. (**B**) Average frequencies and absolute numbers of CD8^+^ and CD4^+^ T cells detected in the lungs of HSV-1-infected and mock-infected control groups. (**C**) Representative FACS plots of the frequencies of gB_498–505_ specific CD8^+^ T cells detected in the lungs of HSV-1 infected and mock-infected control groups. (**D**) Average frequencies and absolute numbers of gB_498–505_ specific CD8^+^ T cells detected in the lungs of HSV-1 infected and mock-infected control groups. The data represent two separate experiments. Graphs depict the mean +/− S.E. of 5 mice/group. * Results were statistically significant at *p* ≤ 0.05.

After observation the innate immune response on day 2, we investigated the adaptive immunity in young (*n* = 5), middle age (*n* = 5), and aged mice (*n* = 5). On day 6 post-infection, the mice were euthanized, and the frequency of lung-resident CD4^+^ and CD8^+^ T cells was determined by flow cytometry (FACS). The overall high frequencies of CD4^+^ and CD8^+^ T cells were induced in the herpes-infected group as compared to the mock-infected group in all three groups of mice, which further declined upon aging (Figure 7A). Average frequencies and absolute numbers of CD8^+^ and CD4^+^ T cells were detected in the lungs of HSV-1-infected and mock-infected control groups (Figure 7B). We next compared the frequency of CD8^+^ T cells specific to HSV-1 gB_498–505_ epitope, using the tetramers/anti-CD8 mAbs, in the lungs of 6-, 36- and 68-week-old mice. The representative dot plots shown in Figure 7C indicate an increased frequency of gB_498–505_-specific CD8^+^ T cells in the lungs of herpes-infected mice, which significantly decreases with aging. Figure 7D shows median frequencies detected in five 6-week-old, 36-week-old, and 68-week-old mice. The lowest frequency of tetramer^+^ CD8^+^ T cells was consistently detected in the aged mice compared to young mice (6.3% vs. 11.9%), indicating that aged mice have an impaired T cell response.

### 3.8. The Cytotoxic Functional Activity of CD8^+^ T Cells Is Reduced with Age in HSV-Infected Mice

**Figure 8:**
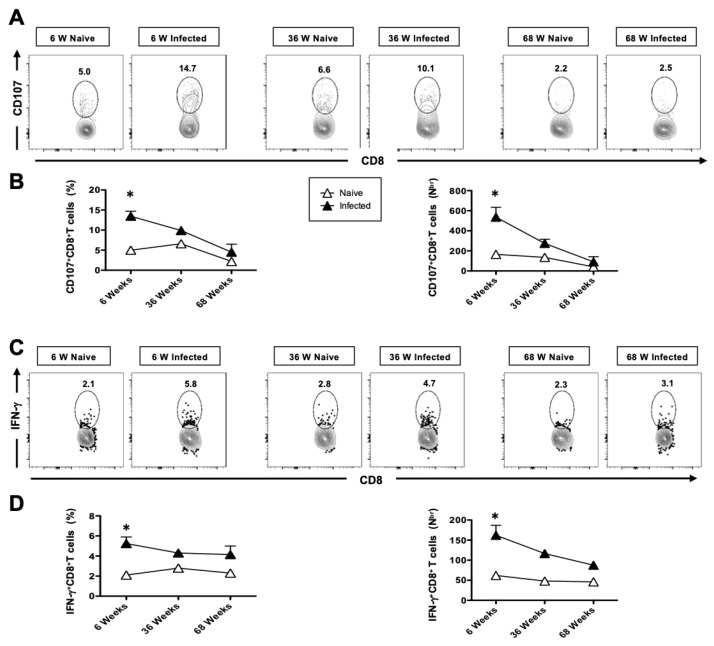
The cytotoxic and functional activity of CD8^+^ T cells detected in HSV-1-infected mice: 6-week-old, 36-week-old, and 68-week-old mice were infected intranasally with 1 × 10^6^ pfu of HSV-1 strain. Seven days post-infection, the mice were euthanized, and a single-cell suspension from the lungs was obtained after collagenase treatment. (**A**) Representative FACS plots of the frequencies of CD107^+^CD8^+^ T cells detected in the lungs of HSV-1-infected and mock-infected control groups. (**B**) Average frequencies and absolute numbers of CD107^+^CD8^+^ T cells detected in the lungs of HSV-1-infected and mock-infected control groups. (**C**) Representative FACS plots of the frequencies of IFN-g^+^CD8^+^ T cells detected in the lungs of HSV-1-infected and mock-infected control groups. (**D**) Average frequencies and absolute numbers of IFN-γ^+^CD8^+^ T cells detected in the lungs of HSV-1-infected and mock-infected control groups. The data is representative of two separate experiments. Graphs depict the mean +/− S.E. of 5 mice/group. Results were statistically significant at *p* ≤ 0.05. represented by “∗”.

The impact of aging on the immune system results in defects in T cell functional responsiveness. To this end, we assessed aging-related T-cell functional response by studying CD107 and IFN-γ by T cells of young and aged mice (*n* = 5). On day 6 post-infection, the mice were euthanized, and a single-cell suspension from the lung tissue was obtained. The function of lung-resident CD8^+^ T cells was then analyzed using flow cytometry (FACS). Significantly high numbers of CD107^+^ CD8^+^ T cells were detected in the lungs of 6-week-old (*n* = 5) and 36-week-old (*n* = 5) HSV-1-infected mice compared to mock-infected mice (Figure 8A,B). However, there was a significant decline in the functional response in 68-week-old mice. As shown in Figure 8C, significantly higher frequencies of functional IFN-γ^+^CD8^+^ T cells were detected in HSV-1-infected mice compared to mock-infected mice (Figure 8D), with a notable decline in these cells observed in the 68-week-old mice. Taken together, these results indicate that HSV-1-infected 6- and 36-week-old mice induced more functional CD8^+^ T cells; however, their presence was significantly impacted in the 68-week-old mice.

## 4. Discussion

The herpes virus affects humans of all ages; however, the elderly (>65 years old) have an increased susceptibility to these infections and are especially predisposed to complications [21,22,23]. The increased morbidity and mortality reported in elderly populations are due to several factors, including dysfunctions in the senescent immune system. Recent studies have shown an increased risk of HSV-1-related diseases in aged individuals. These findings suggest that anti-HSV-1 immunoglobulins in elderly individuals exhibit virus persistence and immune dysfunction [24,25].

Our previous work demonstrated that dendritic cells (DCs) from aged subjects activate the epithelium, making it more permeable to infections [26]. Enhanced basal-level inflammation and cellular activation also promote the dissemination of infections. In summary, we observed that age significantly impacts alveolar epithelial cells (AEC), potentially contributing to age-associated chronic respiratory diseases and infections. AECs not only form a barrier to prevent the entry of infections but also distinguish between innocuous and pathogenic inhalants [27]. These cells express toll-like receptors (TLRs) and other pathogen recognition receptors, enabling them to sense and respond to pathogens. However, there is limited information about the alterations in AECs’ innate immune functions with age. A decrease in ciliary beat capacity and increased secretion of mucin have been reported in the literature [28,29,30,31,32].

In the present study, we evaluated the impact of aging on antiviral immunity, focusing on the roles of dendritic cells (DCs) and macrophages, TLR3 expression, and downstream cytokine responses in susceptibility to HSV infections. We observed that TLR3 expression is significantly decreased in AECs from aged mice, indicating impaired ability to sense viral nucleic acid during viral replication (Figure 1). This may be partially responsible for the increased incidence of other viral infections, such as influenza and, recently, COVID-19 (SARS-CoV-2), in the elderly [33,34]. Thus, we observed that AECs from aged mice displayed increased basal activation during HSV-1 infections.

DCs act as sentinels of the immune response, involving both innate and adaptive immunity. They are mainly comprised of two major subsets, the DCs of myeloid origin and the DCs of lymphoid origin. In the murine lung, the predominant populations include resident CD11b^+^/CD11c^+^ cells, also known as conventional DCs (cDCs). The other APC populations analyzed in these animals represent lung macrophages (CD11b^+^/CD11c^−^) and lymphoid DCs (CD11c^+^ cells). These cells possess specialized antigen-processing capabilities and co-stimulatory molecules that enable their efficient endocytosis and presentation of both endogenous and exogenous antigens, thereby initiating an immune response. DCs detect and respond to pathogens through the expression of pattern recognition receptors (PRRs) [35,36], which recognize conserved molecular patterns of pathogens. The activation of PRR results in the production of cytokines that modulate T cell responses [36,37]. Deficiencies in TLR signaling lead to an increased number of diseases, including sepsis, immunodeficiencies, atherosclerosis, and asthma [7,38].

We evaluated TLR expression on cDCs, macrophages, and lymphoid DCs in the context of aging and observed a reduced surface expression of TLR3 (Figure 2). Earlier studies have reported a similar reduction in TLR expression in whole blood samples and colonic biopsies from older individuals [39], as well as in macrophages and pDCs from aged mice [40,41]. Decreased TLR3 expression in the intestinal epithelium has shown evidence of age-dependent susceptibility to rotavirus infection [42]. Additionally, defects in genes related to the TLR3 pathway are associated with an increased susceptibility to HSV-1 and Varicella-Zoster virus encephalitis [43]. While TLR expression in the lung has not been studied extensively in aging. Our observation of DCs and macrophages from aged mice reveals reduced TLR-3 expression, highlighting the broader implications for increased susceptibility to respiratory viruses in the elderly, including HSV-1 and SARS-CoV-2.

APCs are crucial for recognizing infectious microorganisms in conjunction with DCs, particularly for the surveillance of various tissues. Upon encountering pathogens, DCs migrate to lymph nodes where they present antigens to T-cells, triggering the adaptive immune response. Upon antigen recognition, DCs upregulate several molecules, such as MHC class II, to present antigens to CD4^+^ T cells and provide the necessary signal to fully activate these T cells [44]. In our study, MHC class II up-regulation was not altered in lung macrophages after herpes infection; however, it was upregulated in aged DCs (myeloid and lymphoid) at the basal level, indicative of increased baseline activation of DCs in aging. Our observation is consistent with the earlier reports on reduced pDCs in aging [45,46,47]. In our study, we observed an increased expression of dendritic cell activation markers (MHC-I, MHC-II, and CD80/CD86) in both adult and aged mice compared to young mice. Several reports have demonstrated that aging is associated with immunodeficiency, chronic inflammation, and autoimmunity, leading to increased susceptibility to infections and diminished vaccine efficacy in aged individuals [48]. Therefore, the upregulation of activation marker observed in the present study does not reflect a heightened immune response but rather represents a state of innate immune dysregulation, consistent with age-related alterations in dendritic cell function.

Aging is commonly associated with a decline in T cell function, which contributes to the increased susceptibility to viral infections in the elderly [49,50,51]. However, the role of innate immune responses, particularly the production of interferons (IFN), in antiviral defense mechanisms during aging remains poorly understood. IFN has potent anti-viral properties and subsequently activates both innate and adaptive immunity [52]. Previous studies have reported a decreased secretion of IFN-α by aged pDCs, which are the most potent IFN-producing cells [41,53,54,55,56]. Besides pDCs, the mDCs subset of DCs is also capable of producing a substantial level of IFNs in response to viral infection [53,57].

In this study, we demonstrated that the IFN-α response in lung supernatants was upregulated after herpes infection in both young and adult mice. However, aged mice exhibited impaired secretion of IFNs in response to viral infection. These findings align well with previous reports [41,54,58,59,60,61] demonstrating decreased production of IFN-α in aged subjects. In addition to IFN-α, aged DCs showed deficiencies in the production of IL-1β and IL-22. IL-22 has emerged as a key cytokine involved in regulating host defense and epithelial repair responses during viral infection and resolution [62]. IL-22 synergizes with IL-17 to induce the secretion of antibacterial proteins and chemokines and augments cell proliferation and repair following injury. Survival following a superinfection with *Streptococcus pneumoniae* requires IL-22 [63,64], attributable to the protective effects of IL-22 on pulmonary epithelium. IL-22 has been shown to protect the pulmonary epithelium and reduce inflammation in various models of lung injury [65]. Although the IL-22R is localized to airway epithelium before infection, it is upregulated at parenchymal sites of lung remodeling induced by influenza [62]. Furthermore, treatment of H1N1 (PR8/8/34)-infected mice with IL-22-Fc caused a significant reduction in inflammation, neutrophilia, and lung leak. Importantly, this led to improved health outcomes (reduced weight loss, greater activity scores) and decreased mortality [20]. IL-22 has also been found to prevent apoptosis through the production of anti-apoptotic proteins, such as Bcl-2 and Bcl-1, in a bleomycin model of lung injury [66,67,68]. IL-22 is thus integral in protecting the host against the lung damage caused by viral infections and preventing secondary bacterial infections [19,20]. Our data suggest that the deficiency in IL-22 production in aged DCs could contribute to impaired epithelial repair and increased susceptibility to viral infections.

Additionally, we observed impaired CD8^+^ T cell responses in aged mice following herpes infection. Consistent with the delayed production of the cytokines-IFN-α and IL-22, CD4^+^ and CD8^+^ T cells, HSV-specific CD8^+^ T cells showed delayed infiltration into the lungs of aged animals (Figure 7A,B). In a primary infection, herpes-specific cells may play a more prominent role than non-specifically activated cells. Herpes specific CD8^+^ T cells were assayed using gB_498–505_ immunodominant epitopes. Consistent with our hypothesis, gB_498–505_-specific activated CD8^+^ T cells were consistently higher in young and adult mice compared to aged mice (Figure 7C,D). T cell responsiveness to type I IFN, commonly produced in viral and bacterial infections, is known to be pivotal for the generation of adaptive immune responses. Our findings suggest that the reduced IFN-α response in aged mice may contribute to reduced T cell responses. Furthermore, the upregulation of the functional marker CD107 was delayed in CD8^+^ T cells of aged mice (Figure 8). Our data align with a recent report that demonstrates alterations in the T cell compartment of animals infected with the influenza virus [68,69,70]. T cell responsiveness to type I IFN, a cytokine commonly produced during viral and bacterial infections, is critical for the generation of adaptive immune responses.

To evaluate the functional competence of T cells in young, adult, and aged mice, we also analyzed the production of IFN-γ. Remarkably, the increased number of polyfunctional T cells was maintained in young and adult mice but declined in aged mice. Furthermore, the delayed upregulation of CD107, a functional marker of cytotoxic activity, on CD8^+^ T cells in aged mice supports the hypothesis that aging impairs immune responses, particularly antiviral responses. Accordingly, CD3-mediated stimulation revealed that cytotoxic CD8^+^ T cells were more frequent in the lungs of young and adult mice, and their number declined during aging. Since mature (fully primed) DCs efficiently induce cytokine production by CD8^+^ T cells and the generation of cytotoxic T cells [71], the reduced cytokine production by aged CD8^+^ T cells might be the result of reduced APC activation as suggested by alteration in MHCI and MHCII upregulation, potentially leading to a delayed clearance of the virus. Our data suggest a diminished CD8^+^ T cell response in aged mice following viral infections, such as respiratory syncytial and influenza infections [69,72]. Thus, modulating TLR responses may serve as a beneficial strategy to enhance immune responses in the elderly. Our study offers valuable insights into aging-associated immunosenescence during HSV-1 respiratory infection, which affects both innate and adaptive immune compartments. Impaired early innate responses and weakened adaptive immunity, particularly compromised T cell priming, contribute to the exacerbation of respiratory viral infections in aged hosts. These findings highlight the need for age-specific therapeutic strategies, including the development of more effective vaccination approaches and preventive measures. Additionally, managing comorbidities such as cardiovascular disease, diabetes, and chronic pulmonary disorders is essential in reducing susceptibility to severe respiratory infections in elderly individuals [73].

The aged mouse model has proven to be valuable in determining the effect of aging on the immune responses to HSV-1 infection [41]. In summary, our results demonstrate that HSV infection impairs immunity in aged mice and propagates immune senescence. We also demonstrated that herpes-infected mice exhibited perturbations in naive repertoire more profoundly than those seen in aging alone. A highly diverse T-cell population is critical for protection against pathogens. The diversity of the T cell response to infection is a better correlate of protection than the magnitude of the response (13, 41–43). As little as a 2- to 3-fold reduction in TCR repertoire diversity dramatically impairs antigen-specific responses (44, 45). These defects in T cell function during primary immune responses were identified as a major contributor to immune senescence. Importantly, the changes observed in the T-cell repertoire were accompanied by altered functional responses of CD8^+^ and CD4^+^ T cells in herpes virus-positive animals, which impaired their ability to clear the viral infection. This highlights the crucial roles that CD4^+^ and CD8^+^ T cells play in controlling viral infections in aging populations.

Monocyte/macrophage lineage cells play a crucial role in age-associated changes, particularly within the pulmonary system [74,75]. Although our present study did not focus on macrophage polarization, several reports have validated that macrophages from older mice are more prone to an M1 pro-inflammatory phenotype, characterized by the production of cytokines such as IL-6 and TNFα. Additionally, pro-reparative (M2) cytokine production has been observed to decrease in macrophages from older animals. Age-associated dysfunction of innate immune cells is characterized by reduced chemotaxis, decreased MHC class II expression, and diminished antigen presentation capacity [76,77]. Consequently, aging significantly contributes to impaired immune responses to vaccines and infections and is a key factor in the increased mortality observed in elderly populations.

Cellular senescence is a key mediator of physiological dysfunction in lung aging and plays a central role in many age-related immunological decline [78]. Senescent cells accumulate in the lung microenvironment, contributing to chronic inflammation and tissue remodeling. These cells exhibit a senescence-associated secretory phenotype, releasing pro-inflammatory cytokines, chemokines, and proteases that can disrupt immune homeostasis. As a result, the downregulation of MHC class I and II molecules, reduced expression of type I and II interferons (e.g., IFN-γ, IFN-β), and impaired function of innate immune cells, lung epithelial cells, and stromal cells collectively weaken antigen presentation and immune recognition [79,80]. This suggests that cellular senescence plays a direct role in the decline of immune responses in the aging lung, making it a potential target for therapeutic intervention. Future investigations should explore macrophage polarization and validate senescence markers in HSV-1 lung aging animal models.

It is worth noting that in addition to respiratory infections, herpes simplex virus type 1 (HSV-1) is a major cause of ocular diseases, including herpetic keratitis. Studies have shown that aged mice exhibit increased ocular pathology, accompanied by elevated levels of inflammatory cytokines such as IL-1β and IL-6, as well as the chemokine MIP-2 [81]. These findings emphasize that aging not only impacts susceptibility to respiratory HSV-1 infection but also affects ocular disease outcomes in the context of bacterial endophthalmitis pathology.

In conclusion, with the increasing elderly population and their susceptibility to herpes infection, it is crucial to determine the effects of age on the immune system. Identifying the main groups of cells affected by aging could aid in exploring the underlying mechanisms of the immune alterations. This will help in developing effective vaccines and adjuvants to improve the immune responses in this vulnerable population.

## Data Availability

The original contributions presented in this study are included in the article. Further inquiries can be directed to the corresponding author.

## References

[1] Whitley R.J., Kimberlin D.W., Roizman B. (1998). Herpes simplex viruses. Clin. Infect. Dis..

[2] Corey L., Spear P.G. (1986). Infections with herpes simplex viruses (2). N. Engl. J. Med..

[3] Morgan H.R., Finland M. (1949). Isolation of herpes virus from a case of atypical pneumonia and erythema multiforme exudativum with studies of four additional cases. Am. J. Med. Sci..

[4] Isaacs A., Lindenmann J. (1957). Virus interference. I. The interferon. Proc. R. Soc. Lond. B Biol. Sci..

[5] Gaajetaan G.R., Bruggeman C.A., Stassen F.R. (2012). The type I interferon response during viral infections: A “SWOT” analysis. Rev. Med. Virol..

[6] McNab F., Mayer-Barber K., Sher A., Wack A., O’Garra A. (2015). Type I interferons in infectious disease. Nat. Rev. Immunol..

[7] Cook D.N., Pisetsky D.S., Schwartz D.A. (2004). Toll-like receptors in the pathogenesis of human disease. Nat. Immunol..

[8] Hraiech S., Azoulay E., Papazian L. (2022). Herpesviruses in Critically Ill Patients with ARDS. Encyclopedia of Respiratory Medicine. Encycl. Respir. Med..

[9] Duckworth A., Longhurst H.J., Paxton J.K., Scotton C.J. (2021). The Role of Herpes Viruses in Pulmonary Fibrosis. Front. Med..

[10] De Vos N., Van Hoovels L., Vankeerberghen A., Van Vaerenbergh K., Boel A., Demeyer I., Creemers L., De Beenhouwer H. (2009). Monitoring of herpes simplex virus in the lower respiratory tract of critically ill patients using real-time PCR: A prospective study. Clin. Microbiol. Infect..

[11] Flurkey K., Currer J., Harrison D. (2007). The Mouse in Aging Research. Mouse Biomed. Res..

[12] Zhang X., Chentoufi A.A., Dasgupta G., Nesburn A.B., Wu M., Zhu X., Carpenter D., Wechsler S.L., You S., BenMohamed L. (2009). A genital tract peptide epitope vaccine targeting TLR-2 efficiently induces local and systemic CD8+ T cells and protects against herpes simplex virus type 2 challenge. Mucosal Immunol..

[13] Zhang X., Dervillez X., Chentoufi A.A., Badakhshan T., Bettahi I., Benmohamed L. (2012). Targeting the genital tract mucosa with a lipopeptide/recombinant adenovirus prime/boost vaccine induces potent and long-lasting CD8+ T cell immunity against herpes: Importance of MyD88. J. Immunol..

[14] Steinert E.M., Schenkel J.M., Fraser K.A., Beura L.K., Manlove L.S., Igyarto B.Z., Southern P.J., Masopust D. (2015). Quantifying Memory CD8 T Cells Reveals Regionalization of Immunosurveillance. Cell.

[15] Schenkel J.M., Fraser K.A., Masopust D. (2014). Cutting edge: Resident memory CD8 T cells occupy frontline niches in secondary lymphoid organs. J. Immunol..

[16] Holtzman M.J., Byers D.E., Alexander-Brett J., Wang X. (2014). The role of airway epithelial cells and innate immune cells in chronic respiratory disease. Nat. Rev. Immunol..

[17] Lim H.K., Seppanen M., Hautala T., Ciancanelli M.J., Itan Y., Lafaille F.G., Dell W., Lorenzo L., Byun M., Pauwels E. (2014). TLR3 deficiency in herpes simplex encephalitis: High allelic heterogeneity and recurrence risk. Neurology.

[18] Alexopoulou L., Holt A.C., Medzhitov R., Flavell R.A. (2001). Recognition of double-stranded RNA and activation of NF-kappaB by Toll-like receptor 3. Nature.

[19] Ivanov S., Renneson J., Fontaine J., Barthelemy A., Paget C., Fernandez E.M., Blanc F., De Trez C., Van Maele L., Dumoutier L. (2013). Interleukin-22 reduces lung inflammation during influenza A virus infection and protects against secondary bacterial infection. J. Virol..

[20] Pociask D., Yan X., Kolls J. (2015). IL-22 reduces the pulmonary injury and lethality of influenza infection (CCR4P.201). J. Immunol..

[21] Yoshikawa T.T. (1981). Important infections in elderly persons. West. J. Med..

[22] Curran D., Doherty T.M., Lecrenier N., Breuer T. (2023). Healthy ageing: Herpes zoster infection and the role of zoster vaccination. NPJ Vaccines.

[23] Luginbuehl M., Imhof A., Klarer A. (2017). Herpes simplex type 1 pneumonitis and acute respiratory distress syndrome in a patient with chronic lymphatic leukemia: A case report. J. Med. Case Rep..

[24] Lopatko Lindman K., Weidung B., Olsson J., Josefsson M., Kok E., Johansson A., Eriksson S., Hallmans G., Elgh F., Lövheim H. (2019). A genetic signature including apolipoprotein Eε4 potentiates the risk of herpes simplex-associated Alzheimer’s disease. Alzheimers Dement..

[25] Linard M., Letenneur L., Garrigue I., Doize A., Dartigues J.F., Helmer C. (2020). Interaction between APOE4 and herpes simplex virus type 1 in Alzheimer’s disease. Alzheimers Dement..

[26] Prakash S., Agrawal S., Vahed H., Ngyuen M., Benmohamad L., Gupta S., Agrawal A. (2014). Dendritic cells from aged subjects contribute to chronic airway inflammation by activating bronchial epithelial cells under steady state. Mucosal Immunol..

[27] Gomez M.I., Prince A. (2008). Airway epithelial cell signaling in response to bacterial pathogens. Pediatr. Pulmonol..

[28] Busse P.J., Mathur S.K. (2010). Age-related changes in immune function: Effect on airway inflammation. J. Allergy Clin. Immunol..

[29] Busse P.J., Zhang T.F., Srivastava K., Schofield B., Li X.M. (2007). Effect of ageing on pulmonary inflammation, airway hyperresponsiveness and T and B cell responses in antigen-sensitized and -challenged mice. Clin. Exp. Allergy.

[30] Ho J.C., Chan K.N., Hu W.H., Lam W.K., Zheng L., Tipoe G.L., Sun J., Leung R., Tsang K.W. (2001). The effect of aging on nasal mucociliary clearance, beat frequency, and ultrastructure of respiratory cilia. Am. J. Respir. Crit. Care Med..

[31] Svartengren M., Falk R., Philipson K. (2005). Long-term clearance from small airways decreases with age. Eur. Respir. J..

[32] Quiros-Roldan E., Sottini A., Natali P.G., Imberti L. (2024). The Impact of Immune System Aging on Infectious Diseases. Microorganisms.

[33] Bartleson J.M., Radenkovic D., Covarrubias A.J., Furman D., Winer D.A., Verdin E. (2021). SARS-CoV-2, COVID-19 and the Ageing Immune System. Nat. Aging.

[34] Liu Z.M., Yang M.H., Yu K., Lian Z.X., Deng S.L. (2022). Toll-like receptor (TLRs) agonists and antagonists for COVID-19 treatments. Front. Pharmacol..

[35] Ngo C., Garrec C., Tomasello E., Dalod M. (2024). The role of plasmacytoid dendritic cells (pDCs) in immunity during viral infections and beyond. Cell Mol. Immunol..

[36] Iwasaki A., Medzhitov R. (2010). Regulation of adaptive immunity by the innate immune system. Science.

[37] Manicassamy S., Pulendran B. (2009). Modulation of adaptive immunity with Toll-like receptors. Semin. Immunol..

[38] Li D., Wu M. (2021). Pattern recognition receptors in health and diseases. Signal Transduct. Target. Ther..

[39] Rosenstiel P., Derer S., Till A., Hasler R., Eberstein H., Bewig B., Nikolaus S., Nebel A., Schreiber S. (2008). Systematic expression profiling of innate immune genes defines a complex pattern of immunosenescence in peripheral and intestinal leukocytes. Genes. Immun..

[40] Murciano C., Yanez A., O’Connor J.E., Gozalbo D., Gil M.L. (2008). Influence of aging on murine neutrophil and macrophage function against Candida albicans. FEMS Immunol. Med. Microbiol..

[41] Stout-Delgado H.W., Yang X., Walker W.E., Tesar B.M., Goldstein D.R. (2008). Aging impairs IFN regulatory factor 7 up-regulation in plasmacytoid dendritic cells during TLR9 activation. J. Immunol..

[42] Pott J., Stockinger S., Torow N., Smoczek A., Lindner C., McInerney G., Backhed F., Baumann U., Pabst O., Bleich A. (2012). Age-dependent TLR3 expression of the intestinal epithelium contributes to rotavirus susceptibility. PLoS Pathog..

[43] Sironi M., Peri A.M., Cagliani R., Forni D., Riva S., Biasin M., Clerici M., Gori A. (2017). TLR3 Mutations in Adult Patients With Herpes Simplex Virus and Varicella-Zoster Virus Encephalitis. J. Infect. Dis..

[44] Grewal I.S., Flavell R.A. (1998). CD40 and CD154 in cell-mediated immunity. Annu. Rev. Immunol..

[45] Agrawal A., Tay J., Ton S., Agrawal S., Gupta S. (2009). Increased reactivity of dendritic cells from aged subjects to self-antigen, the human DNA. J. Immunol..

[46] Panda A., Qian F., Mohanty S., van Duin D., Newman F.K., Zhang L., Chen S., Towle V., Belshe R.B., Fikrig E. (2010). Age-associated decrease in TLR function in primary human dendritic cells predicts influenza vaccine response. J. Immunol..

[47] Reitsema R.D., Kumawat A.K., Hesselink B.C., van Baarle D., van Sleen Y. (2024). Effects of ageing and frailty on circulating monocyte and dendritic cell subsets. NPJ Aging.

[48] Agrawal A., Agrawal S., Gupta S. (2007). Dendritic cells in human aging. Exp. Gerontol..

[49] Effros R.B. (2004). Replicative senescence of CD8 T cells: Potential effects on cancer immune surveillance and immunotherapy. Cancer Immunol. Immunother..

[50] Gupta S., Bi R., Su K., Yel L., Chiplunkar S., Gollapudi S. (2004). Characterization of naive, memory and effector CD8+ T cells: Effect of age. Exp. Gerontol..

[51] Haynes L., Maue A.C. (2009). Effects of aging on T cell function. Curr. Opin. Immunol..

[52] Randall R.E., Goodbourn S. (2008). Interferons and viruses: An interplay between induction, signalling, antiviral responses and virus countermeasures. J. Gen. Virol..

[53] Diebold S.S., Montoya M., Unger H., Alexopoulou L., Roy P., Haswell L.E., Al-Shamkhani A., Flavell R., Borrow P., Reis e Sousa C. (2003). Viral infection switches non-plasmacytoid dendritic cells into high interferon producers. Nature.

[54] Jing Y., Shaheen E., Drake R.R., Chen N., Gravenstein S., Deng Y. (2009). Aging is associated with a numerical and functional decline in plasmacytoid dendritic cells, whereas myeloid dendritic cells are relatively unaltered in human peripheral blood. Hum. Immunol..

[55] Sridharan A., Esposo M., Kaushal K., Tay J., Osann K., Agrawal S., Gupta S., Agrawal A. (2011). Age-associated impaired plasmacytoid dendritic cell functions lead to decreased CD4 and CD8 T cell immunity. Age.

[56] Psarras A., Alase A., Antanaviciute A., Carr I.M., Md Yusof M.Y., Wittmann M., Emery P., Tsokos G.C., Vital E.M. (2020). Functionally impaired plasmacytoid dendritic cells and non-haematopoietic sources of type I interferon characterize human autoimmunity. Nat. Commun..

[57] Katashiba Y., Miyamoto R., Hyo A., Shimamoto K., Murakami N., Ogata M., Amakawa R., Inaba M., Nomura S., Fukuhara S. (2011). Interferon-alpha and interleukin-12 are induced, respectively, by double-stranded DNA and single-stranded RNA in human myeloid dendritic cells. Immunology.

[58] Shodell M., Siegal F.P. (2002). Circulating, interferon-producing plasmacytoid dendritic cells decline during human ageing. Scand. J. Immunol..

[59] Canaday D.H., Amponsah N.A., Jones L., Tisch D.J., Hornick T.R., Ramachandra L. (2010). Influenza-induced production of interferon-alpha is defective in geriatric individuals. J. Clin. Immunol..

[60] Qian F., Wang X., Zhang L., Lin A., Zhao H., Fikrig E., Montgomery R.R. (2011). Impaired interferon signaling in dendritic cells from older donors infected in vitro with West Nile virus. J. Infect. Dis..

[61] Feng E., Balint E., Poznanski S.M., Ashkar A.A., Loeb M. (2021). Aging and Interferons: Impacts on Inflammation and Viral Disease Outcomes. Cells.

[62] Pociask D.A., Scheller E.V., Mandalapu S., McHugh K.J., Enelow R.I., Fattman C.L., Kolls J.K., Alcorn J.F. (2013). IL-22 is essential for lung epithelial repair following influenza infection. Am. J. Pathol..

[63] McAleer J.P., Kolls J.K. (2014). Directing traffic: IL-17 and IL-22 coordinate pulmonary immune defense. Immunol. Rev..

[64] Mills K.H.G. (2023). IL-17 and IL-17-producing cells in protection versus pathology. Nat. Rev. Immunol..

[65] Yi P., Liang Y., Yuan D.M.K., Jie Z., Kwota Z., Chen Y., Cong Y., Fan X., Sun J. (2017). A tightly regulated IL-22 response maintains immune functions and homeostasis in systemic viral infection. Sci. Rep..

[66] Sonnenberg G.F., Nair M.G., Kirn T.J., Zaph C., Fouser L.A., Artis D. (2010). Pathological versus protective functions of IL-22 in airway inflammation are regulated by IL-17A. J. Exp. Med..

[67] Jiang J., Fisher E.M., Murasko D.M. (2011). CD8 T cell responses to influenza virus infection in aged mice. Ageing Res. Rev..

[68] Cadar A.N., Martin D.E., Bartley J.M. (2023). Targeting the hallmarks of aging to improve influenza vaccine responses in older adults. Immun. Ageing.

[69] Effros R.B., Walford R.L. (1983). The immune response of aged mice to influenza: Diminished T-cell proliferation, interleukin 2 production and cytotoxicity. Cell Immunol..

[70] Parks O.B., Eddens T., Sojati J., Lan J., Zhang Y., Oury T.D., Ramsey M., Erickson J.J., Byersdorfer C.A., Williams J.V. (2023). Terminally exhausted CD8(+) T cells contribute to age-dependent severity of respiratory virus infection. Immun. Ageing.

[71] Larsson M., Messmer D., Somersan S., Fonteneau J.F., Donahoe S.M., Lee M., Dunbar P.R., Cerundolo V., Julkunen I., Nixon D.F. (2000). Requirement of mature dendritic cells for efficient activation of influenza A-specific memory CD8+ T cells. J. Immunol..

[72] Fulton R.B., Weiss K.A., Pewe L.L., Harty J.T., Varga S.M. (2013). Aged mice exhibit a severely diminished CD8 T cell response following respiratory syncytial virus infection. J. Virol..

[73] de Miguel-Díez J., Núñez Villota J., Santos Pérez S., Manito Lorite N., Alcázar Navarrete B., Delgado Jiménez J.F., Soler-Cataluña J.J., Pascual Figal D., Sobradillo Ecenarro P., Gómez Doblas J.J. (2024). Multidisciplinary Management of Patients With Chronic Obstructive Pulmonary Disease and Cardiovascular Disease. Arch. Bronconeumol..

[74] Oishi Y., Manabe I. (2016). Macrophages in age-related chronic inflammatory diseases. NPJ Aging Mech. Dis..

[75] Kim I.H., Kisseleva T., Brenner D.A. (2015). Aging and liver disease. Curr. Opin. Gastroenterol..

[76] Jackaman C., Tomay F., Duong L., Abdol Razak N.B., Pixley F.J., Metharom P., Nelson D.J. (2017). Aging and cancer: The role of macrophages and neutrophils. Ageing Res. Rev..

[77] Chen S., Saeed A.F.U.H., Liu Q., Jiang Q., Xu H., Xiao G.G., Rao L., Duo Y. (2023). Macrophages in immunoregulation and therapeutics. Signal Transduct. Target. Ther..

[78] Torrance B.L., Haynes L. (2022). Cellular senescence is a key mediator of lung aging and susceptibility to infection. Front. Immunol..

[79] Schafer M.J., White T.A., Iijima K., Haak A.J., Ligresti G., Atkinson E.J., Oberg A.L., Birch J., Salmonowicz H., Zhu Y. (2017). Cellular senescence mediates fibrotic pulmonary disease. Nat. Commun..

[80] Zhou L., Ruscetti M. (2023). Senescent macrophages: A new “old” player in lung cancer development. Cancer Cell.

[81] Singh P.K., Singh S., Wright R.E., Rattan R., Kumar A. (2020). Aging, But Not Sex and Genetic Diversity, Impacts the Pathobiology of Bacterial Endophthalmitis. Invest. Ophthalmol. Vis. Sci..

